# Relationship among DDR gene mutations, TMB and PD-L1 in solid tumour genomes identified using clinically actionable biomarker assays

**DOI:** 10.1038/s41698-023-00442-4

**Published:** 2023-10-11

**Authors:** Danyi Wang, Brian Elenbaas, Karthikeyan Murugesan, Kunal Shah, Meagan Montesion, Ioannis Gounaris, Juergen Scheuenpflug, Giuseppe Locatelli, Zheng Feng

**Affiliations:** 1 Clinical Measurements Sciences, Global Research & Development, EMD Serono Research & Development Institute, Inc., an affiliate of Merck KGaA, Billerica, MA USA; 2 Research Unit Oncology, EMD Serono Research & Development Institute, Inc., an affiliate of Merck KGaA, Billerica, MA USA; 3 Foundation Medicine, Cambridge, MA USA; 4 Global Clinical Development, Merck Serono Ltd., an affiliate of Merck KGaA, Feltham, UK; 5 Clinical Measurements Sciences, Global Research & Development, Merck Healthcare KGaA, Darmstadt, Germany

**Keywords:** Tumour biomarkers, Cancer genomics

## Abstract

The DNA damage response (DDR) pathway regulates DNA repair and cell survival, and inactivating mutations in DDR genes can increase tumour mutational burden (TMB), a predictive biomarker of treatment benefit from anti-PD-1/PD-L1 immunotherapies. However, a better understanding of the relationship among specific DDR mutations, TMB and PD-L1 expression is needed to improve translational strategies. Here, we determined genomic alteration frequencies in selected DDR genes that are clinically actionable biomarkers and investigated their association with TMB and PD-L1 in bladder, colorectal, non-small cell lung, ovarian and prostate cancers using the FoundationInsights^®^ web portal. Our results not only confirm known associations, such as mismatch repair and *POLE* gene mutations with high TMB, but also identify significant associations between mutations in the SWI/SNF chromatin remodelling genes *ARID1A* and *SMARCA4* and high TMB in multiple tumour types. Mutations in the *ATR* gene were associated with high TMB in colorectal and prostate cancers; however, associations between individual DDR mutations and high PD-L1 expression were uncommon and tumour-type specific. Finally, we found that high TMB and high PD-L1 expression were poorly associated, emphasising their independence as predictive biomarkers for immune checkpoint inhibitor use.

## Introduction

Genomic instability has long been recognised as a cancer-enabling characteristic^1,2^. DNA damage response (DDR) pathways and repair mechanisms play a key role in preserving human genomic stability, and as they encompass multiple processes and components, their activation depends on the type of DNA damage and phase of the cell cycle^3,4^. Defective DNA repair due to aberrations in DDR components plays a critical role in tumour genomic instability, thereby resulting in cancer development and progression^1,2,5^.

Given their role in cancer, there is significant interest in DDR genes as prognostic markers, predictors of response to therapy and targets for therapy^3,4^. Genomic alterations (GA) in genes involved in the homologous recombination (HR) DNA damage repair pathway, such as the BRCA1/2 pathway, result in homologous repair deficiency and can be used to identify patients with tumours that may be sensitive to treatment with agents such as poly(ADP-ribose) polymerase (PARP) inhibitors in breast, ovarian (OC), prostate (PC) and pancreatic cancers. Additionally, these GAs may enhance tumour cell sensitivity to DNA-damaging chemotherapeutic drugs, such as platinum analogues and temozolomide^6–8^. GAs in HR genes, other than those in *BRCA1/2*, have been identified in many cancers, and preclinical studies suggest that specific HR GAs confer sensitivity to inhibitors of other DDR genes, such as ataxia-telangiectasia mutated (ATM), ataxia-telangiectasia and RAD3-related (ATR) and DNA-dependent protein kinase^9^. Furthermore, the tumour DNA repair landscape may play an important role in determining the response to immune checkpoint blockade^10^ because patients harbouring tumours with extensive DNA damage have been shown to respond better to immunotherapy^11^. These observations indicate the potential benefit of further exploring the role of DDR GAs and the use of recognised predictive biomarkers of immunotherapy to appropriately stratify patients based on tumour mutational burden (TMB), microsatellite instability (MSI) and PD-L1 expression^12–14^. Furthermore, available evidence suggests that combining DDR inhibitors with immunotherapy may have synergistic effects in tumours harbouring DDR GAs^10,15^.

Comprehensive genomic profiling (CGP) is being increasingly used for routine clinical management of patients with cancer^16^ and several studies have used CGP data to examine the potential of GAs and genomic instability signatures to inform targeted treatment in various cancers. For instance, in a study of approximately 3500 PC samples, 57% harboured GAs that are investigational biomarkers for targeted therapies^17^. In another study, 17% of ~17,500 gastrointestinal tumours had alterations in one or more of the 10 DDR genes that are known therapeutic targets^18^ and 21% of tumours with DDR GAs had high TMB, supporting the potential use of a combination of DDR inhibition with immunotherapy^18^. Furthermore, biallelic *BRCA1/2* alterations were associated with increased genomic loss of heterozygosity (gLOH) in diverse solid tumours^19^, and biallelic HR GAs, other than those in *BRCA1/2*, were associated with increased genomic scar scores^20^. Thus, data from these and other studies point towards novel strategies for patient selection that will benefit from utilising genomics-based DDR mutational biomarkers for DDR inhibition and immunotherapy.

Although the consequences of DDR deficiency are becoming increasingly clear, significant gaps in knowledge remain, warranting further studies on the therapeutic impact of individual DDR GAs. As identifying associations between these DDR GAs and other biomarkers (e.g., TMB and PD-L1) and signatures of genomic instability (e.g., MSI and gLOH) is expected to have an increasingly important role in biomarker-driven precision oncology, we selected 35 DDR genes from the FoundationOne^®^ gene panel in which somatic and germline mutations leading to genomic instability and increased mutation rates have been documented across a range of cancers^1,21,22^. These 35 genes can be grouped into eight DNA damage and response functions or pathways: base excision repair, damage sensor, Fanconi anaemia, HR, mismatch repair, nucleotide-excision repair, SWI/SNF chromatin remodelling and TP53 pathway (Table 1). Except for *MDM2* and *MDM4* from the TP53 pathway, which are oncogenes that can acquire copy number gains in certain cancers, the selected DDR genes are tumour suppressor genes that can undergo inactivating mutations or copy number loss in cancer. SWI/SNF chromatin remodelling genes play many roles, including regulation of chromatin assembly, transcription and DNA repair, and several of these genes are important tumour suppressor genes^23^. Here, we investigated the two most frequently mutated SWI/SNF genes, *ARID1A* and *SMARCA4*. Further, we focused our study on five tumour types (bladder cancer [BC], colorectal cancer [CRC], non-small cell lung cancer [NSCLC], OC and PC), to permit exploration of any association between DDR GAs and clinically relevant immunotherapy biomarkers (PD-L1, TMB) and identification of potential opportunities for combination therapy. Thus, this analysis was designed to provide comprehensive molecular profiling data on DDR deficiency in selected advanced solid tumours for which the immunotherapy is currently, (i) an established treatment option in most or all patients (BC, NSCLC), (ii) not generally indicated, but used in certain subgroups, such as MSI-high (MSI-H) or TMB-high (CRC, OC, PC).

**Table 1:** The 35 DDR genes analysed and their function in repair.

Base excision repair	Damage sensor	Fanconi anaemia	Homologous recombination		Mismatch repair	Nucleotide excision repair	SWI/SNF chromatin remodelling	TP53 pathway
*MUTYH*	*ATM*	*FANCA*	*BARD1*	*RAD51*	*MLH1*	*ERCC4*^a^	*ARID1A*	*MDM2*
*PARP1*^a^	*ATR*	*FANCC*	*BRCA1*	*RAD51B*^a^	*MSH2*	*POLE*	*SMARCA4*	*MDM4*
	*CHEK1*	*FANCG*	*BRCA2*	*RAD51C*^a^	*MSH3*^a^			*TP53*
	*CHEK2*	*FANCL*	*BRIP1*	*RAD51D*^a^	*MSH6*			
		*XRCC2*^a^	*NBN*^a^	*RAD52*^a^	*PMS2*			
			*PALB2*	*RAD54L*^a^				

^a^Included only in the FoundationOne^®^CDx assay. All other genes were included in both the FoundationOne^®^CDx and FoundationOne^®^ assays.

## Results

### Clinical characteristics and genomic features of the patient population

To assess the prevalence of GAs in DDR genes, we analysed CGP results for tumours from 159,638 patients: 7803 with BC; 44,646 with CRC; 70,496 with NSCLC; 21,209 with OC; and 15,484 with PC (Table 2). The relative numbers and demographics were as expected for patients with solid tumours. TMB-high status was more prevalent in NSCLC (n = 24,510; 34.8%) and BC (*n* = 2611; 33.5%) than in CRC (*n* = 3876; 8.7%), PC (*n* = 748; 4.8%), or OC (*n* = 709; 3.3%; Table 2). Among patients with PD-L1 data (Table 2), the prevalence of PD-L1-high status was 52.8% in BC (*n* = 1344), 33.2% in OC (*n* = 2065), 32.4% in NSCLC (*n* = 9434), 13.2% in PC (*n* = 486) and 5.1% in CRC (*n* = 1656). The use of different cut-offs for PD-L1-high status in different tumour types precluded any comparison of these data.

**Table 2:** Patient clinical characteristics and tumour genomic features.

	Bladder cancer	CRC	NSCLC	Ovarian cancer	Prostate cancer
Patients (n)	7803	44,646	70,496	21,209	15,484
Median age, years (IQR)	70 (62–77)	60 (51–69)	68 (60–75)	62 (54–70)	67 (61–74)
Patient-reported sex, n (%)					
Male	5709 (73.2)	24,497 (54.9)	35,005 (49.7)	1 (0.0)	15472 (99.9)
Female	2091 (26.8)	20,124 (45.1)	35,465 (50.3%)	21,208 (100.0)	4 (0.0)
Unknown	3 (0.0)	25 (0.0)	26 (0.0)	0	8 (0.0)
Tumour biopsy site					
Local	4636	22,799	37,340	6352	8519
Metastatic	2519	18,239	26,422	10,718	5876
Unknown	648	3608	6734	4139	1089
Median TMB, mut/Mb (IQR)	6.25 (3.75–12.18)	3.75 (1.74–5.22)	6.25 (2.6–12.5)	2.5 (1.25–4.35)	1.74 (1.25–3.75)
TMB-H cases^a^, n (%)	2611 (33.5)	3876 (8.7)	24,510 (34.8)	709 (3.3)	748 (4.8)
PD-L1-H cases/n with PD-L1 data^b^, n (%)	1344/2545 (52.8)	1656/10,982 (5.1)	9434/29,156 (32.4)	2065/6220 (33.2)	486/3683 (13.2)
TMB-H and PD-L1-H (n)	538	333	3656	103	52
TMB-H and PD-L1-low (n)	361	704	6734	125	121
TMB-low and PD-L1-H (n)	806	1323	5777	1962	434
TMB-low and PD-L1-low (n)	840	8622	12,984	4030	3076
(*p* value)	(*p* = 1.61E–07)	(*p* = 3.14E–48)	(*p* = 2.93E–05)	(*p* = 1.4E–04)	(*p* = 2.35E–09)

*CRC* colorectal cancer, *IQR* interquartile range, *mut/Mb* mutations/Mb, *NSCLC* non-small cell lung cancer, *PD-L1-H* PD-L1-high, *TMB* tumour mutational burden, *TMB-H* TMB-high.

^a^High TMB (TMB ≥ 10 mut/Mb).

^b^Cut-off: DAKO 22C3 Tumour Stain, score cut-off = 1 for CRC, ovarian cancer and prostate cancer; score cut-off = 10 for bladder cancer; score cut-off = 50 for NSCLC.

### DDR mutational landscape across tumour types

**Fig. 1:**
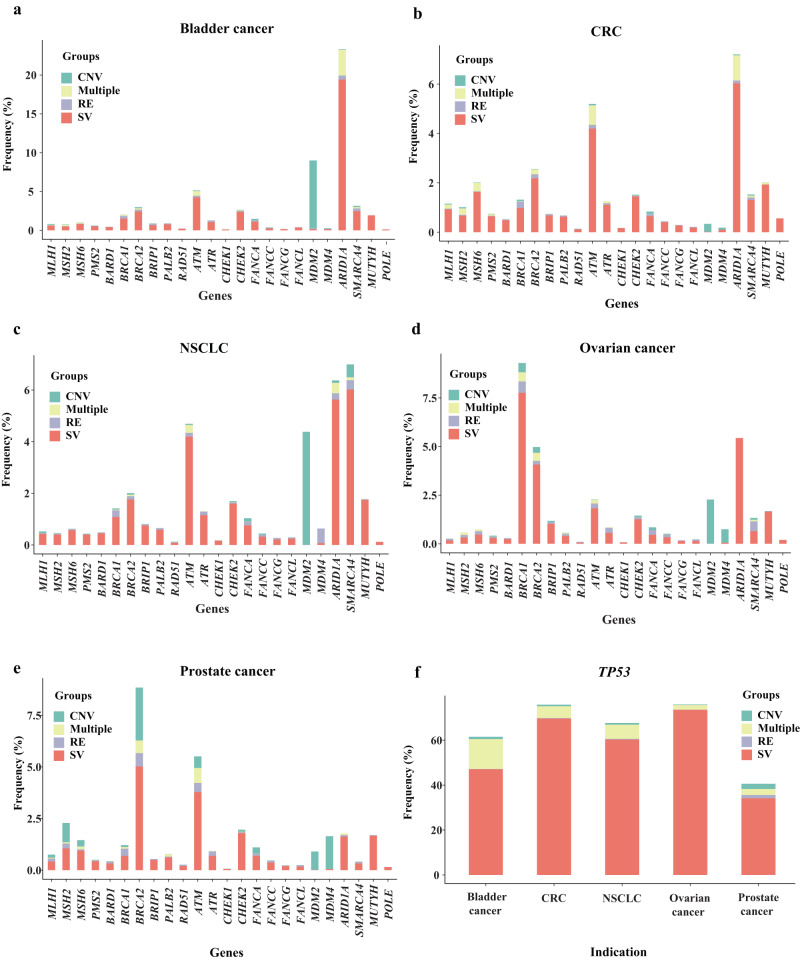
DDR GA landscape across tumour types. DDR GAs were frequent and non-uniformly distributed based on type and frequency across cancer types (**a**–**e**). *TP53* (**f**) was the most frequently altered DDR gene across all cancers (68.1%), followed by *ARID1A* (7.2%), *ATM* (4.6%), *SMARCA4* (3.9%), *MDM2* (2.9%), *BRCA2* (3.3%), *BRCA1* (2.4%), *MUTYH* (1.8%), *CHK2* (1.7%) and *MSH6* (1.1%), whereas many other DDR genes were rarely altered (≤1%). SVs in DNA repair genes were assessed for function. For each gene, the number of cases with a single SV only, CNV only, RE only, or multiple GAs is shown. CRC colorectal cancer, CNV copy number variation, DDR DNA damage response, GA genomic alteration, NSCLC non-small cell lung cancer, RE rearrangement, SV short variant.

Analysis of the DDR mutational landscape showed that DDR GAs were common across tumour types but that they were non-uniformly distributed according to variant type and frequency (Fig. 1a–e, Table 3, Supplementary Fig. 1). As expected, *TP53* (Fig. 1f, Table 3) was the most frequently altered DDR gene across all five tumour types (68.1%), followed by *ARID1A* (7.2%), *ATM* (4.6%), *SMARCA4* (3.9%), *MDM2* (3%), *BRCA2* (3.3%), *BRCA1* (2.4%), *MUTYH* (1.8%), *CHEK2* (1.7%), *ATR* (1.2%), and *MSH6* (1.1%). The TP53 pathway-related gene *MDM2* was commonly amplified in BC (8.7%; copy number variant [CNV] 8.72%, short variant [SV] 0.14%) and NSCLC (4.40%; CNV 4.38%, SV 0.0014%). GAs in the other DDR genes were rare (≤1.0%).

**Table 3:** Frequency of DDR GAs across tumour types.

Gene class	Gene	Bladder cancer	CRC	NSCLC	Ovarian cancer	Prostate cancer	Average frequency across all 5 tumours analysed^b^
Base excision repair	*MUTYH*	1.94	2.02	1.78	1.69	1.69	1.83
	*PARP1*^a^	0.07	0.06	0.23	0.42	0.07	0.11
Damage sensor	*ATM*	5.14	5.19	4.69	2.28	5.51	4.61
	*ATR*	1.28	1.26	1.31	0.85	0.93	1.19
	*CHEK1*	0.12	0.18	0.18	0.08	0.06	0.15
	*CHEK2*	2.63	1.52	1.70	1.45	1.96	1.69
Fanconi anaemia	*FANCA*	1.46	0.84	1.04	0.85	1.10	0.98
	*FANCC*	0.36	0.45	0.44	0.52	0.46	0.45
	*FANCG*	0.17	0.30	0.27	0.18	0.23	0.26
	*FANCL*	0.40	0.22	0.29	0.23	0.24	0.26
	*XRCC2*^a^	0	0.05	0.02	0.02	0.03	0.02
Homologous recombination	*BARD1*	0.45	0.53	0.49	0.30	0.44	0.47
	*BRCA1*	2.00	1.32	1.41	9.30	1.21	2.44
	*BRCA2*	3.00	2.56	2.00	5.00	8.84	3.26
	*BRIP1*	0.88	0.74	0.81	1.18	0.54	0.82
	*NBN*^a^	0.87	1.10	0.92	0.45	0.93	0.57
	*PALB2*	0.90	0.68	0.66	0.56	0.77	0.67
	*RAD51*	0.22	0.14	0.12	0.10	0.26	0.14
	*RAD51B*^a^	0.60	0.34	0.31	0.32	0.38	0.21
	*RAD51C*^a^	0.27	0.26	0.24	0.62	0.27	0.19
	*RAD51D*^a^	0.21	0.26	0.27	0.53	0.14	0.18
	*RAD52*^a^	0	0.01	0.01	0	0	0.00
	*RAD54L*^a^	0.39	0.31	0.27	0.29	0.36	0.19
Mismatch repair	*MLH1*	0.81	1.16	0.52	0.27	0.75	0.70
	*MSH2*	0.74	1.01	0.46	0.58	2.28	0.82
	*MSH3*^a^	0.67	3.22	0.67	0.59	1.24	0.89
	*MSH6*	1.00	2.01	0.63	0.74	1.45	1.13
	*PMS2*	0.60	0.74	0.44	0.42	0.50	0.53
Nucleotide excision repair	*ERCC4*^a^	0.25	0.24	0.21	0.17	0.29	0.14
	*POLE*	0.12	0.57	0.12	0.21	0.15	0.26
SWI/SNF chromatin remodelling	*ARID1A*	23.31	7.20	6.37	8.30	1.78	7.24
	*SMARCA4*	3.14	1.53	6.99	1.33	0.42	3.89
TP53 pathway	*MDM2*	8.74	0.34	4.39	2.28	0.90	2.85
	*MDM4*	0.26	0.18	0.63	0.75	1.64	0.60
	*TP53*	61.45	75.78	67.66	75.89	40.58	68.09

*DDR* DNA damage response, *GA* genomic alteration, *CRC* colorectal cancer, *NSCLC* non-small cell lung cancer.

^a^Included only in the FoundationOne^®^CDx assay. All other genes were included in both the FoundationOne^®^CDx and FoundationOne^®^ assays.

^b^Frequency in samples with ≥1 DDR gene mutation (%).

In terms of GA based on DDR function, GAs in the following genes were relatively uncommon across tumour types: mismatch repair (<3.0%), Fanconi anaemia (<2%), and base– and nucleotide–excision repair (<2.5%). In contrast, HR genes were frequently altered in OC (*BRCA1*, 9.3%; *BRCA2*, 5.0%) and PC (*BRCA2*, 8.8%). Alterations in DNA damage sensor genes were also prevalent across tumour types, with *ATM* mutations found in ~5% of BC, NSCLC, CRC and PC specimens. Furthermore, GAs related to SWI/SNF chromatin remodelling were frequent in BC (*ARID1A*, 23.3%), NSCLC (*ARID1A*, 6.4%; *SMARCA4*, 7.0%), CRC (*ARID1A*, 7.2%) and OC (*ARID1A*, 8.3%) but were rare in PC ( < 2.5%).

SV mutations were the most common GA type across tumour types and genes (Fig. 1); specifically, SV mutations accounted for 47.1% of the *TP53* GAs in BC, 69.7% in CRC, 60.3% in NSCLC, 73.5% in OC and 34.2% in PC (Fig. 1f). As expected, the TP53 pathway genes *MDM2* and *MDM4* almost exclusively harboured gene CNVs.

### Distribution of germline and somatic status of SV mutations in DDR genes

**Fig. 2:**
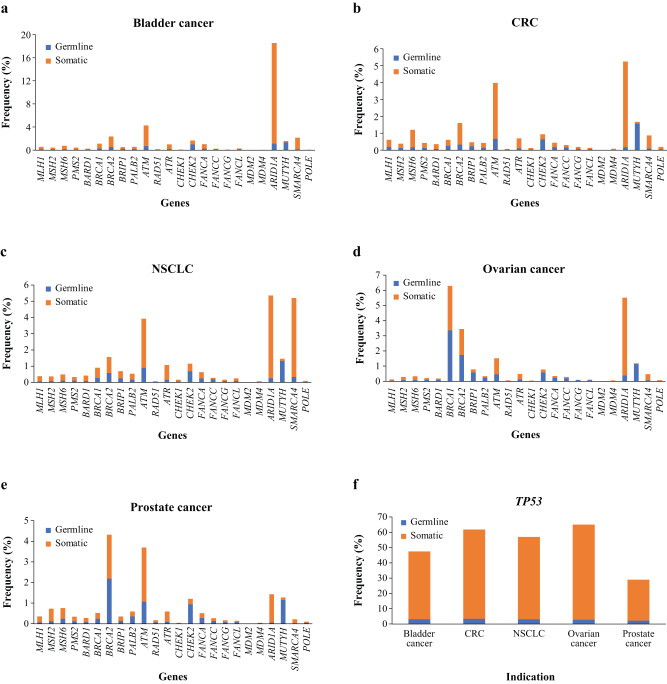
SVs in DNA repair genes assessed for germline-only or somatic-only prevalence. SV mutations were assessed for germline- or somatic-only status (**a**–**e**). Across the tumour types analysed, SV mutations of somatic origin were predominant in TP53 (**f**). CRC colorectal cancer, NSCLC non-small cell lung cancer, SV short variant.

SV mutations were assessed for germline– or somatic–only status. Across the tumour types analysed, SV mutations of somatic origin were predominant in *TP53* (Fig. 2f**;** BC, 44.4%; CRC, 58.4%; NSCLC, 53.9%; OC, 62.2%; PC, 26.9%), the DNA damage sensor gene *ATM* (BC, 3.6%; CRC, 3.3%; NSCLC, 3.0%; OC, 1.1%; PC, 2.6%), SWI/SNF chromatin remodelling genes *ARID1A* (BC, 17.4%; CRC, 5.1%; NSCLC, 5.1%; OC, 5.1%; PC, 1.4%), *SMARCA4* (BC, 2.0%; CRC, 0.8%; NSCLC, 4.9%; OC, 0.4%; PC, 0.2%) and in the mismatch repair gene *MSH3* (BC, 0.3%; CRC, 1.6%; NSCLC, 0.4%; OC, 0.2%; PC, 0.6%; Supplementary Fig. 2). The proportion of germline SV mutations in *BRCA1* and *BRCA2* was markedly elevated in OC (*BRCA1*, 3.4%; *BRCA2*, 1.7%; Fig. 2d) and PC (*BRCA2*, 2.2%; Fig. 2e) compared to other cancers, as was the proportion of germline SV mutations in the HR genes *RAD51C* and *RAD51D* in OC (Supplementary Fig. 2). In addition, germline mutations were primarily identified (Fig. 2a–e) in *MUTYH* (BC, 1.3%; CRC, 1.5%; NSCLC, 1.3%; OC, 1.1%; PC, 1.2%) and *CHEK2* (BC, 1.0%; CRC, 0.6%; NSCLC, 0.7%; OC, 0.6%; PC, 1.0%).

### DDR GA type and relationship with TMB

**Fig. 3:**
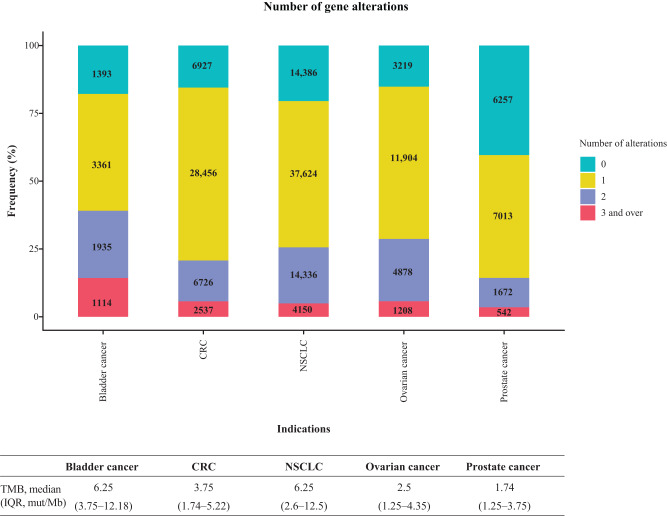
Number of GAs in 25 DDR genes (included in both the FoundationOne^®^CDx and FoundationOne^®^ assays). 0: no alteration in DDR genes; 1: one alteration in DDR genes, 2: two alterations in DDR genes, >3: at least three alterations in DDR genes (could be same gene or multiple different genes). CRC colorectal cancer, DDR DNA damage response, GA genomic alteration, IQR interquartile range, NSCLC non-small cell lung cancer, TMB tumour mutational burden.

We identified a sizeable proportion of tumours with multiple DDR GAs in the TMB-high tumour type BC and the highest proportion of tumours without DDR GAs in the TMB-low tumour type PC (Fig. 3 and Supplementary Fig. 3). This suggests that, in some cases, DDR GAs may be a consequence of a TMB-high phenotype. However, in some cases, such as the TMB-low tumour type OC, the highest proportion of tumours with one or more DDR GAs was observed, arguing against a simple relationship between TMB-high status and multiple GAs (Fig. 3 and Supplementary Fig. 3). However, for each tumour type, a weak but significant positive correlation was observed between the number of DDR GAs and TMB (BC: Spearman *ρ* = 0.308; CRC: Spearman *ρ* = 0.172; NSCLC: Spearman *ρ* = 0.358; OC: Spearman *ρ* = 0.261; PC: Spearman *ρ* = 0.206; all *p* < 2.2E–16).

Median TMB based on GA type (CNV, rearrangement and SV) was also assessed (Table 4). As noted above, SVs were the most common type of GA across tumour types, and this was also true for the tumours with available TMB data (range 50.7–84.1%). PC, which had the lowest median TMB overall (1.74) (Table 2), had the lowest proportion of SVs (50.7%) but a high proportion of rearrangements (4.8%) and CNVs (9.5%) compared with the other tumour types. However, median TMB was similar for PC with each of these GA types, as was the case for CRC. In contrast, OC, which also had a relatively low median TMB (2.5), had the highest proportion of SVs (84.1%) and CRC tumours with rearrangements had a higher median TMB than those with SV or CNVs. In BC and NSCLC, the median TMB for tumours with CNVs was lower than that for the other two GA types. Overall, no apparent relationship between the proportion of tumours with the different GA types and median TMB was observed.

**Table 4:** Median TMB according to GA type.

Tumour type	Number with TMB data	Alteration type	Number with alteration (%)	Median TMB (interquartile range)
Bladder cancer	7803	CNV	864 (11.1)	6.2 (3.5–11.2)
		Rearrangement	246 (3.2)	7.8 (5.0–13.8)
		SV	5935 (76.1)	7.5 (3.8–13.0)
CRC	44,645	CNV	774 (1.7)	3.8 (2.5–6.2)
		Rearrangement	677 (1.5)	3.8 (2.5–6.2)
		SV	37,216 (83.4)	3.8 (1.7–6.1)
NSCLC	70,486	CNV	4831 (6.9)	5 (2.5–10.0)
		Rearrangement	1614 (2.3)	7.8 (3.8–13.9)
		SV	53,171 (75.4)	7.8 (3.8–13.9)
Ovarian cancer	21,207	CNV	1001 (4.7)	2.6 (1.2–5.0)
		Rearrangement	818 (3.9)	3.8 (1.7–6.1)
		SV	17,837 (84.1)	2.5 (1.2–4.3)
Prostate cancer	15,483	CNV	1477 (9.5)	2.6 (1.2–5.2)
		Rearrangement	745 (4.8)	2.5 (1.2–3.8)
		SV	7846 (50.7)	2.5 (1.2–3.8)

*CNV* copy number variation, *TMB* tumour mutational burden, *GA* genomic alteration, *SV* short variant, *CRC* colorectal cancer, *NSCLC* non-small cell lung cancer.

### Relationship between TMB and alterations in individual DDR genes

**Fig. 4:**
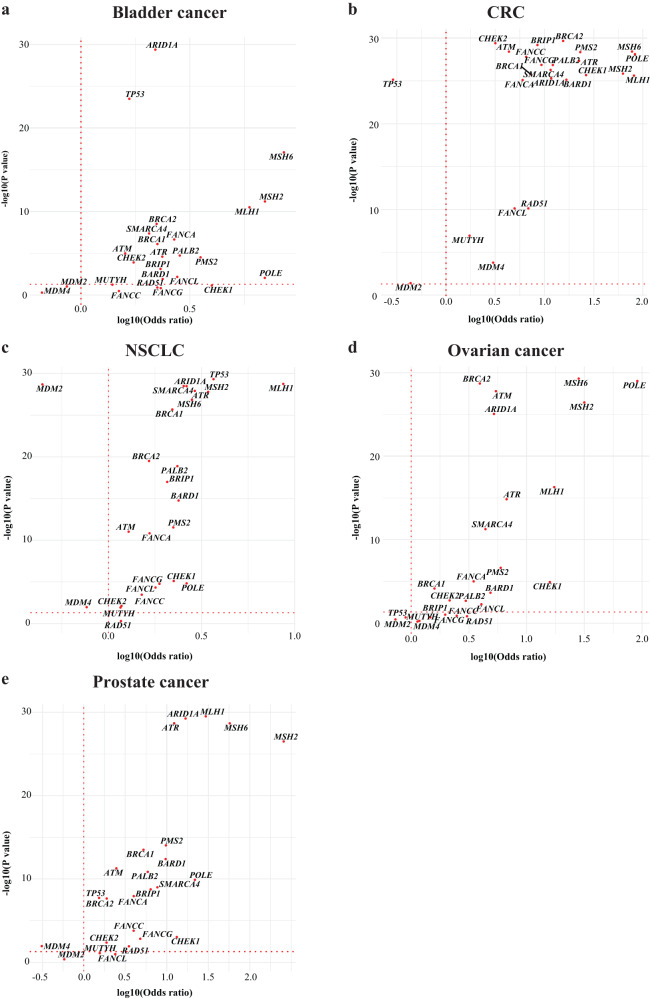
Relationship between TMB and 25 DDR GAs. For each tumour type (**a**–**e**), Fisher’s exact test was used to estimate the odds ratios and *p* values. *P* values were adjusted for multiple testing using the Benjamini–Hochberg method. Only genes with an adjusted *p* value ≤ 5% are labelled. For visualisation purposes, genes with a *p* value < 10^−25^ had their *p* values randomised between 10^−25^ and 10^−30^. A log10(odds ratio) >0 indicates an association with high TMB (TMB ≥ 10 mut/Mb) and a log10(odds ratio) <0 indicates an association with low TMB. The red dotted vertical line depicts an odds ratio of 1, implying no association. CRC colorectal cancer, DDR DNA damage response, GA genomic alteration, mut/Mb mutations/Megabase, NSCLC non-small cell lung cancer, TMB tumour mutational burden.

Individual GAs were plotted for each tumour type using Fisher’s exact test to estimate the odds ratios (ORs) and *p* values (Fig. 4a–e). As this analysis revealed that certain genes displayed particularly significant associations with TMB, we focused on genes with a log_10_(*p* value) of >20 and a log_10_(OR) of >0.5 and found significant associations between mutations in multiple mismatch repair genes and TMB in all tumour types (CRC: *MLH1*, *MSH2*, *MSH6* and *PMS2*; NSCLC: *MLH1* and *MSH2*; OC: *MSH2* and *MSH6*; PC: *MLH1*, *MSH2* and *MSH6*), except BC. We also found significant associations between mutations in the nucleotide excision repair gene *POLE* and TMB in CRC and OC. The SWI/SNF chromatin remodelling gene *ARIDIA* was significantly associated with TMB in CRC, OC and PC and *SMARCA4* mutations were significantly associated with TMB in CRC. Finally, significant associations of *ATR* mutations, a DNA damage sensor gene, with TMB were observed in CRC and PC (Fig. 4b, e). In CRC, significant associations of HR (*BRCA1*, *BRCA2*, *BARD1*, *PALB2* and *BRIP1*) and Fanconi anaemia (*FANCA*, *FANCC* and *FANCG*) genes with TMB were identified. This suggests a CRC-specific link between these repair processes and TMB.

### Relationship between PD-L1 and alterations in individual DDR genes

We undertook an exploratory analysis of DDR GAs and PD-L1 protein expression using immunohistochemistry (IHC) data for PD-L1 obtained using the Dako 22C3 assay. For PD-L1, a tumour proportion scoring (TPS) threshold of 1% was used for all tumour types, except BC, wherein a combined positive score (CPS) threshold of 10 was used, and NSCLC, wherein a TPS threshold of 50% was used. PD-L1 data were available for 32.6%, 24.7%, 42.4%, 29.3% and 23.8% of the BC, CRC, NSCLC, OC and PC specimens, respectively.

**Fig. 5:**
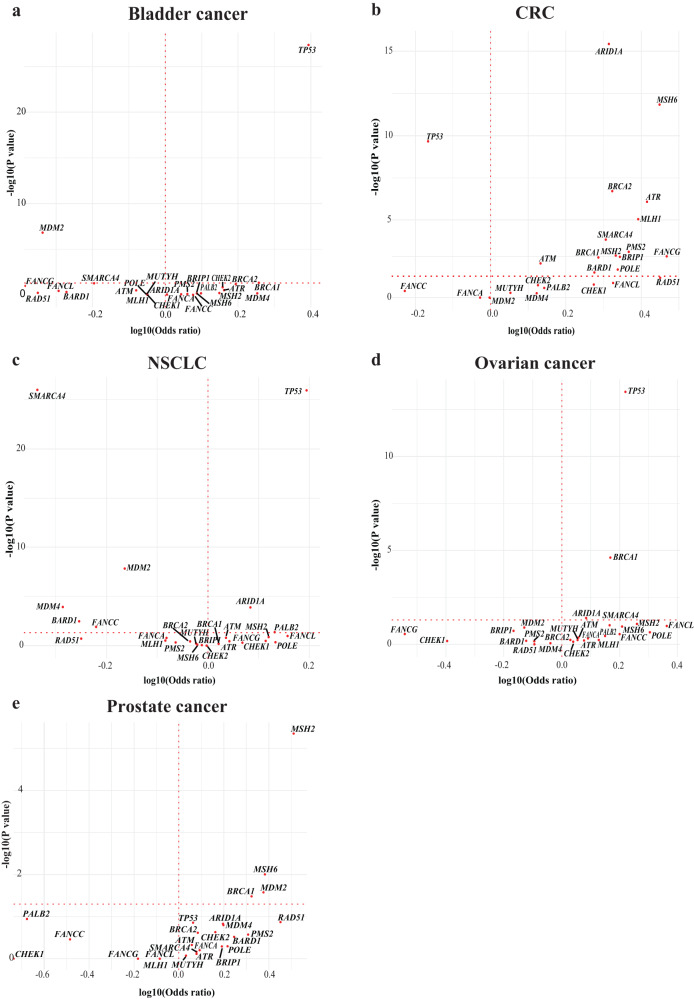
Relationship between PD-L1 and DDR GAs. For each tumour type (**a**–**e**), Fisher’s exact test was used to estimate the odds ratios and *p* values. *p* values were adjusted for multiple testing using the Benjamini–Hochberg method. A log10(odds ratio) >0 indicates an association with high PD-L1 and a log10(odds ratio) <0 indicates an association with low PD-L1. The red dotted vertical line depicts an odds ratio of 1, implying no association. CRC colorectal cancer, DDR DNA damage response, GA genomic alteration, NSCLC non-small cell lung cancer.

In contrast to the interesting associations observed between certain DDR GAs and TMB, significant associations between DDR GAs and PD-L1-high status were uncommon (Fig. 5a–e). *TP53* mutations were associated with PD-L1-high status in BC (OR 2.48, *p* = 5.48E–26), NSCLC (OR = 1.56, *p* = 7.2E–58) and OC (OR 1.65, *p* = 4.66E–13) but not in CRC or PC. The only other strong associations between genes and PD-L1-high status that were observed in these five tumour types were for *ARID1A* (OR 2.06, *p* = 9.43E–15) and *MSH6* (OR 2.81, *p* = 1.86E–11) in CRC and *MSH2* in PC (OR 3.24, *p* = 1.16E–4). No significant associations between genes grouped according to DDR pathway and PD-L1-high status were observed.

### Overlap of TMB-high and PD-L1-high status and correlation with DDR GAs

**Fig. 6:**
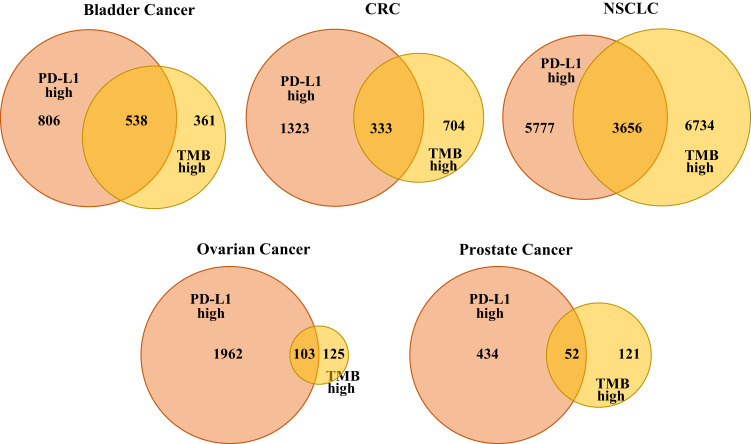
Overlap of TMB-high and PD-L1-high statuses. CRC colorectal cancer, NSCLC non-small cell lung cancer, TMB tumour mutational burden.

**Fig. 7:**
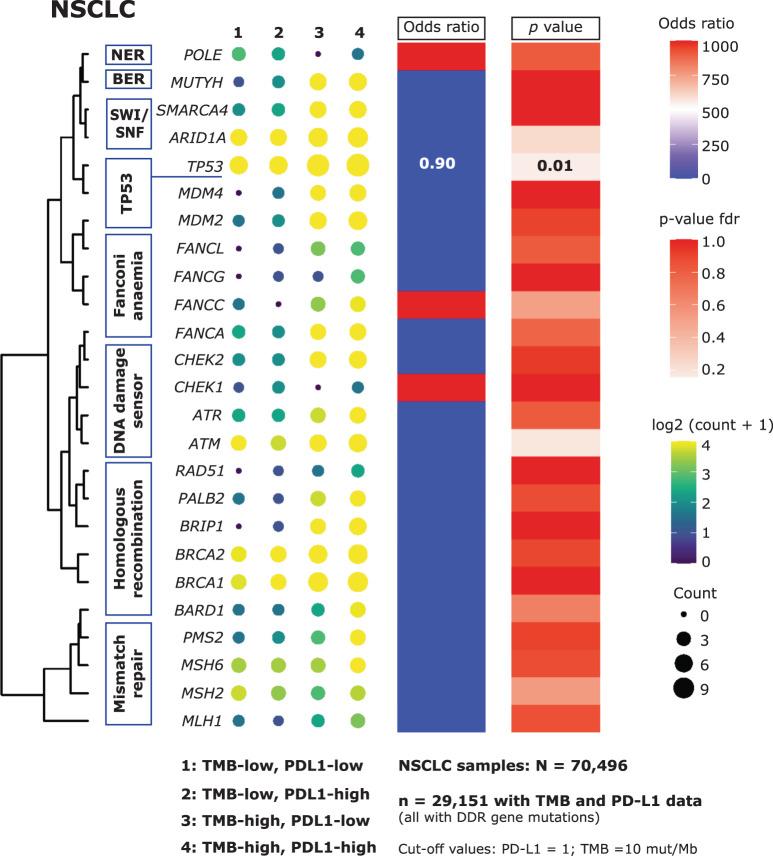
Correlation of DDR GA with TMB-high or PD-L1-high status in NSCLC samples. To represent variations among different patient populations based on their TMB and PD-L1 mutation status, the number of samples with gene mutation detected are annotated using size and colour. Both the colour and size of the dots are proportional to the count of each gene. For example, in TMB-high and PD-L1 high population, there are 5673 patients with *TP53* mutations; in TMB-high and PD-L1 low population, there are 3082 patients with *TP53* mutations; in TMB-low and PD-L1 high population, there are 7743 patients with *TP53* mutations; in TMB-low and PD-L1 low population, there are 3802 patients with *TP53* mutations. A negative relationship between *TP53* mutation and TMB-high/PD-L1-high tumours (OR 0.90, *p* = 1.3E–3) was observed in NSCLC. The odds ratio comes from the Fisher’s exact test on c(5673, 3082, 7743, 3802). In the *TP53* mutants, PDL1-high is depleted in the TMB-high group—(5673)/(5673 + 3082) when compared to the TMB-low group—(7743)/(7743 + 3802). The cluster represents a group of genes that exhibit patterns in correlation to TMB and PD-L1 status. BER base excision repair, DDR DNA damage response, GA genomic alteration, NER nucleotide excision repair, NSCLC non-small cell lung cancer, SWI/SNF SWI/SNF chromatin remodelling, TMB tumour mutational burden.

In general, the reported correlation between TMB-high and PD-L1-high statuses is low. In this study, the proportion of TMB-high tumours that were also PD-L1-high differed across tumour types (Fig. 6). Overlap was the highest in the TMB-high BC (59.8% of TMB-high tumours were also PD-L1-high) and OC (45.2%), followed by NSCLC (35.2%), CRC (32.1%) and PC (30.1%). In tumours that were both TMB-high and PD-L1-high, a significant enrichment of GAs in individual DDR genes was observed in CRC (*ARID1A*: OR 4.39, *p* = 4.56E–17; *TP53*: OR 2.67, *p* = 3.01E–17; *ATR*: OR 7.55, *p* = 8.0E–4; *ATM*: OR 3.69, *p* = 5.95E–07; *BRCA2*: OR 4.10, *p* = 5.42E–05) and PC (*TP53*: OR 3.81, *p* = 4.07E–06). In NSCLC, we observed a negative relationship between *TP53* mutation and TMB-high/PD-L1-high tumours (OR 0.90, *p* = 1.3E–3; Fig. 7). No significant relationships between individual DDR genes and TMB-high/PD-L1-high status were found in BC or OC.

## Discussion

We used the FoundationOne^®^ and FoundationOne^®^CDx assays to characterise GAs in 35 DDR genes for which somatic and germline mutations have been documented^21,22^ and to examine the association of these GAs with TMB and PD-L1 status in 159,638 solid tumours. Our data indicate that most tumours harbour GAs in at least one DDR gene (~60%–85% depending on tumour type) and that GAs are often seen in more than one DDR gene (~15–40% depending on tumour type). GAs in DDR genes varied according to type and frequency across the tumour types investigated, with *TP53* being the most frequently altered across all five tumour types. We have previously described the molecular profiles of 269,371 clinically advanced and diverse malignancies, and while that data set included samples from 14 types of malignancies^24^, this report utilised data from five tumour types and represents an extended and in-depth analysis of the same.

GAs were relatively common in SWI/SNF chromatin remodelling genes *ARID1A* and *SMARCA4*, which are important tumour suppressor genes^23^, the TP53 pathway-related gene *MDM2*, the DNA damage sensors *ATM* and *ATR*, and *BRCA1* and *BRCA2*, whereas GAs in other DDR genes were rare. Furthermore, while the overall frequencies of GAs in the genes studied were comparable to those reported previously^17–19,25,26^, higher rates of mutations in the SWI/SNF chromatin remodelling genes have been reported^27^. Although GAs in mismatch repair, Fanconi anaemia, base excision repair and nucleotide excision repair genes were relatively uncommon, HR genes were frequently altered in OC and PC. Mutations in *MUTYH*, a base excision repair gene involved in correcting DNA errors resulting from guanine oxidation, were also relatively common. In a retrospective analysis of next-generation sequencing data, pathogenic mutations in the nucleotide excision repair gene *POLE* were associated with a clinical benefit from immune checkpoint inhibition therapy^28^. In our study, significant associations were observed between *POLE* mutations and TMB in CRC and OC. SV mutations were the most common type of GA across all tumour types and genes.

The objective of this study was to identify potential and clinically relevant associations between DDR GAs and TMB or PD-L1 status. The incidence of TMB-high (defined as ≥10 mutations per megabase [mut/Mb]) and PD-L1-high (score cut-off = TPS 1 for CRC, OC and PC; CPS 10 for BC; and TPS 50 for NSCLC) tumours differed among cancer types. Although the cut-offs used to assess TMB and PD-L1 statuses vary in the literature, the observed incidences of TMB-high and PD-L1-high tumours were generally similar to those reported previously^29–37^. Our data showed that TMB-high and PD-L1-high statuses in each of the five tumour types were poorly correlated. Overlap was the highest in NSCLC and BC, and PD-L1-targeted therapies are approved for both^38–43^. The TMB cut-off of 10 mut/Mb was based on a previous study on NSCLC^44^; however, emerging evidence suggests that this cut-off maybe too broad and additional insights can be gained from examining different cut-offs for different tumour types^45–48^.

Immune checkpoint inhibitors, such as pembrolizumab, nivolumab, avelumab, atezolizumab and durvalumab, are not only approved for use in patients with tumours that express PD-L1 but are also indicated for certain solid tumour types without the need to assess PD-L1 expression^38–43,49–54^. Furthermore, while pembrolizumab and nivolumab are indicated for use in patients with MSI-H or mismatch repair-deficient solid tumours, both in the USA and in Europe^39,40,49,50^, pembrolizumab is indicated for use in patients with TMB-high solid tumours in the USA^49^. A pan-cancer study indicated that TMB-high status predicts response to anti-PD-1 therapy, independent of PD-L1 status, attesting to the independent predictive value of these biomarkers^55,56^ and suggesting that GAs associated with TMB-high status may have clinical relevance.

Our data indicate that while most cancers harbour GAs in at least one DDR gene (~60%–85% depending on tumour type), the frequency of cancers with DDR GAs does not correlate with median TMB (e.g., 84.8% of OCs had at least 1 DDR GA but a median TMB of 2.5 mut/Mb), indicating that the relationship between DDR GA and TMB is complex. For example, in the relationship between individual DDR GAs and TMB-high tumour status, CRC, OC and PC specimens were enriched with multiple DDR GAs, particularly in genes involved in nucleotide excision repair, mismatch repair, SWI/SNF chromatin remodelling and sensing DNA damage. We hypothesise that GAs in these genes are more likely to result in SVs and, eventually, in high TMB, whereas HR mutations result in larger rearrangements and CNVs, which are not captured by TMB. In contrast, BC and NSCLC showed high TMB but had either no enrichment of specific DDR GAs (BC) or only a few enriched DDR GAs (NSCLC). One explanation could be that TMB-high status in BC and NSCLC is the result of damage caused by exogenous carcinogens, such as tobacco smoke, a known aetiologic factor for both tumour types^57,58^, rather than being primarily driven by early clonal DDR defects.

Average CNV has previously been identified as an indicator of high TMB and immunotherapy efficacy in NSCLC, and we found a relatively high proportion of CNVs in NSCLC and BC, which are both tumour types with high median TMB. Notably, a similar association between TMB, CNVs and DNA methylation was observed in a study on Chinese patients, but it highlighted the need for further research to identify TMB thresholds for personalised lung cancer immunotherapy in different patient populations^59^. We also report a relatively high proportion of CNVs in PC, which has low median TMB and for which immunotherapy has shown limited efficacy. No association between CNVs and TMB was observed in CRC or OC, which are also tumour types for which immunotherapy has shown limited efficacy^60–62^. The significance of these findings and the role of TMB versus CNVs in determining sensitivity to immunotherapy warrants further investigation.

The DNA damage sensor genes *ATM* and *ATR* encode key kinases that help orchestrate DDR. Although both kinases are activated by DNA damage and replication stress, *ATM* acts as a regulator of cell cycle checkpoints and double-strand break repair, whereas *ATR* regulates cell cycle progression and promotes fork repair to overcome replication stress^63^. Of note, the high median TMB tumour types, NSCLC and BC, were particularly enriched with GAs in *ATM*. It has been shown that ATM-deficient cells have higher levels of type I interferon-stimulated genes (ISGs) and that ATR inhibition of these cells further increases ISG levels, resulting in dendritic cell activation^64^. Thus, tumours with *ATM* GAs may be sensitive to a synthetic lethality approach with ATR inhibitors, with or without the addition of immune checkpoint inhibitors.

We also found that *ARID1A* was commonly altered in BC, NSCLC, CRC and OC and that TMB-high tumours were enriched with *ARID1A* GAs. Further, as cancer cell models deficient in ARID1A are more sensitive to ATR inhibition through a synthetic lethality mechanism^65^, there is a rationale to combine immunotherapy with ATR inhibitors in TMB-high tumours harbouring *ARID1A* GAs, which is based on data linking *ARID1A* GAs and TMB-high status to ATR inhibitor and immune checkpoint inhibitor sensitivity, respectively.

The association of mutations in the SWI/SNF chromatin remodelling genes, *ARID1A* and *SMARCA4*, as well as *ATR* with high TMB represent a testable clinical hypothesis. The association of clinical responses to immune checkpoint inhibitors and SWI/SNF mutations may be further evaluated in other tumour types using larger clinico-genomic cohorts to determine whether these mutations could serve as additional biomarkers for immune checkpoint inhibitor therapy. The association between mutations in *ATR* with high TMB also imply that ATR mutations can lead to high TMB and a subsequent high neoantigen burden, warranting clinical investigation into the efficacy of combination treatment with ATR inhibitors and immune checkpoint inhibitors. However, it is possible that mutations in the SWI/SNF family, particularly *ARID1A* mutations that co-occur in high TMB tumours, are bystander mutations of the high TMB/MSI mutation phenotype, and thus, may not be causally related. It has been recently shown that MSI-H cancers often contain *BRCA1/2* mutations; however, these mutations are predominantly monoallelic and are considered bystander alterations because they are not associated with gLOH and did not confer PARP sensitivity in several patients^66^. Arguing against the concept that SWI/SNF and *ATR* mutations are merely bystander mutations of MSI-H is the fact that all the mutations in this study were classified as functional or pathogenic mutations. Therefore, these DDR gene mutation associations warrant further investigations into their allelic status, functional relevance and potential clinical utility.

Our results also indicate that tumour type-dependent selection of the correct DDR gene for biomarker studies and the relevant DDR inhibitor and immune checkpoint inhibitor for combination trial strategies will be essential. For example, we show that GAs in genes such as *ARID1A*, *SMARCA4* and *ATR* are associated with TMB-high status in a tumour type-specific manner and may thus represent additional biomarkers for immunotherapy in these tumour types. Furthermore, our findings suggest that the combination of immune checkpoint inhibitors and ATR inhibitors to which tumours with the aforementioned GAs are sensitive to in preclinical models and early clinical data, warrants further investigation.

To provide maximum sensitivity, a PD-L1 IHC score of TPS ≥ 1% was used as an exploratory cut-off for indications without a CDx claim in this study, namely, PC, OC and CRC. One of the limitations of using PD-L1 IHC TPS ≥ 1% as the cut-off in the tumour types without a CDx indication is that there is a lack of data on clinical outcomes in patients treated with immunotherapy using this PD-L1 cut-off; therefore, this analysis was considered exploratory^67^. In addition, TMB-high has been previously shown to be a biomarker of response in mismatch repair-deficient CRC, albeit at a higher TMB threshold. We acknowledge that the lack of mismatch repair status is a limitation of this study^68^.

In conclusion, these results, derived using clinically available and actionable assays, advance our understanding of the association between DDR GAs in cancer, and support the investigation of immune checkpoint inhibitors, not only in tumour type-specific and biomarker-defined subgroups (e.g., mutated *ARID1A, SMARCA4, ATR*) but also in combination with ATR inhibitors, in the clinical setting.

## Methods

### Comprehensive genomic profiling (CGP)

CGP of clinical solid tumour cases was performed in a Clinical Laboratory Improvement Amendments (CLIA)-certified and College of American Pathologists (CAP)-accredited laboratory using the FoundationOne^®^ and FoundationOne^®^CDx assays (Foundation Medicine, Inc., Cambridge, MA, USA) as previously described^69,70^. The data were derived from de-identified and research-consented cases across five different solid tumour types, namely, BC, CRC, NSCLC, OC and PC, and were profiled between August 2014 and July 2021.

Formalin-fixed paraffin-embedded specimens containing at least 20% tumour cells and yielding a minimum of 50 ng of extracted DNA were subjected to hybridisation-captured, adapter ligation-based library preparation to identify GAs (SVs), CNVs and rearrangements in all coding exons (FoundationOne^®^CDx: *n* = 309 genes; FoundationOne^®^: *n* = 395 genes) and selected introns (FoundationOne^®^CDx: *n* = 36 genes; FoundationOne^®^: *n* = 31 genes) in cancer-associated genes. Further analysis was performed on cases with GAs in any of the 35 genes selected based on an adequate representation of the DDR pathways and coverage by the Foundation Medicine assay. Of these, 25 DDR-associated genes were baited across both FoundationOne^®^CDx and FoundationOne^®^ assays, while 10 DDR-associated genes were baited only on the FoundationOne^®^CDx assay (Table 1). The predicted somatic or germline status of the GAs was determined using previously described methods^19,71^. TMB was calculated as the number of non-driver somatic coding mut/Mb of the genome sequenced; TMB-high was defined as ≥10 mut/Mb and TMB-low as <10 mut/Mb^72^. Briefly, for each sample, we created a genome-wide copy number profile with circular binary segmentation and a Gibbs sampling Markov Chain Monte Carlo algorithm, on the basis of log-ratios to a process-matched control and allele frequencies at over 3500 genome-wide single nucleotide polymorphisms^70,71^. Germline/somatic mutation calls were predicted without a matched normal. Germline/somatic calls for an alteration were estimated by modelling the alteration’s allele frequency, taking into account tumour content, tumour ploidy, and local copy number. In validation testing of 480 tumour-only sequencing calls against matched normal samples, the accuracy was 95% for somatic and 99% for germline calls.^71^

Only GAs (SVs, CNVs, or rearrangements) that are described as functional or pathogenic in literature and are listed in the Catalogue of Somatic Mutations in Cancer repository or had a likely functional status (e.g. frameshift or truncation events in tumour suppressor genes) were included in this study^73^. Variants of unknown significance were excluded.

PD-L1 IHC analysis was performed and interpreted by experienced board-certified pathologists according to the manufacturer’s instructions in a CLIA-certified and CAP-accredited laboratory (Foundation Medicine, Morrisville, North Carolina, USA) for a subset of specimens in this cohort using DAKO PD-L1 IHC 22C3 pharmDx^74^. A TPS (proportion of PD-L1-stained tumour cells) method was used to score all tumour types (score cut-off of 1 for CRC, OC and PC, and 50 for NSCLC), except for BC, in which the combined positive scoring (CPS; proportion of PD-L1-stained tumour, lymphocyte and macrophage cells) method was used (score cut-off of 10), as previously reported^67^.

The prevalence of detected GAs in the 35 DDR genes were categorised according to gene and disease, including base substitutions, small indels, CNVs and rearrangements. TMB scores and PD-L1 expression data were retrieved from the Foundation Medicine FoundationInsights^®^ web platform. Data on tumour site and patient age at the time of CGP were extracted from accompanying pathology reports, clinical notes and test order requisition forms.

### Statistical analyses

All statistical analyses were performed using the R (v4.0.3) and Python (v2.7.6) software packages. For each tumour type, Fisher’s exact test was used to estimate ORs and *p* values for the relationships between biomarkers (TMB or PD-L1) and DDR GAs. All *p* values were two-sided and multiple hypothesis testing correction was performed using the Benjamini–Hochberg procedure to calculate false discovery rate. Spearman’s correlation and univariate linear regression were used to determine the association between the number of DDR GAs (across 35 DDR genes, including multiple GAs per gene, if present) per specimen and the TMB score for each tumour type.

### Reporting summary

Further information on research design is available in the Nature Research Reporting Summary linked to this article.

### Supplementary information

Supplementary material

Reporting summary

## Data Availability

The authors declare that all relevant aggregate data supporting the findings of this study are available within the article and its supplementary information files. In accordance with the Health Insurance Portability and Accountability Act, we do not have IRB approval or patient consent to share individualised patient genomic data, which contains potentially identifying or sensitive patient information and cannot be reported in a public data repository. Foundation Medicine is committed to collaborative data analysis and has well-established and widely used mechanisms by which qualified researchers can query their core genomic database of >500,000 de-identified sequenced cancers. More information and mechanisms for data access can be obtained by contacting the corresponding author or the Foundation Medicine Data Governance Council at data.governance.council@foundationmedicine.com.
