# Supplementary material for: Relationship among DDR gene mutations, TMB and PD-L1 in solid tumour genomes identified using clinically actionable biomarker assays
Source: NPJ Precis Oncol. 2023 Oct 11;7:103. doi: 10.1038/s41698-023-00442-4 (PMC10567713; doi:10.1038/s41698-023-00442-4)
Supplement: Supplementary file 1 — Supplementary material [file 41698_2023_442_MOESM1_ESM.pdf]

Supplementary materials

Supplementary figure 1: DDR GA landscape across the tumour types for genes included in the FoundationOne®CDx assay

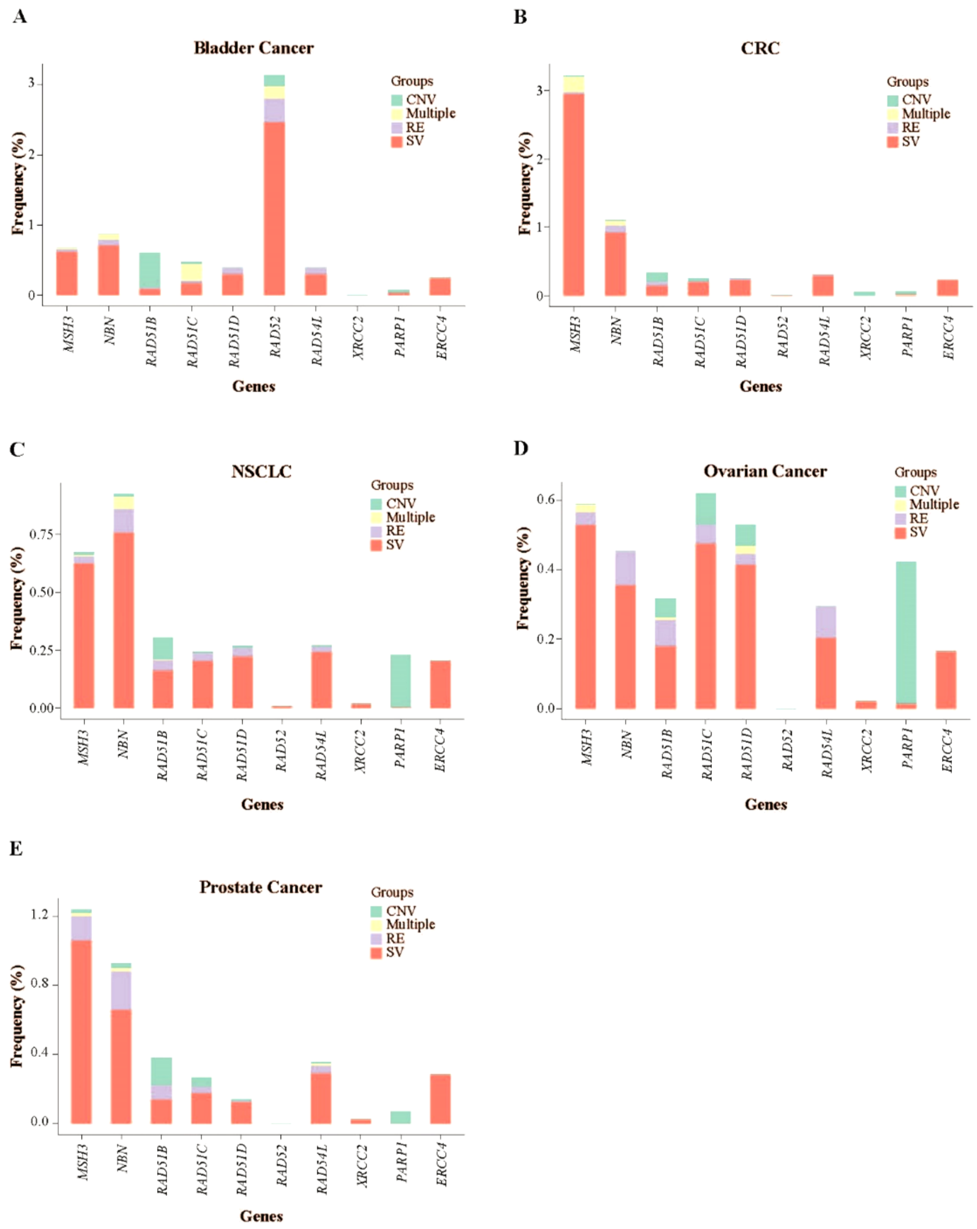

DDR GAs were frequent and non-uniformly distributed based on type and frequency across the tumour types. SVs in DNA repair genes were assessed for function. For each gene, the number of cases with a single SV only, CNV only, RE only, or multiple GAs is shown (Panels A–E). CNV, copy number variation; CRC, colorectal cancer; DDR, DNA damage response; GA, genomic alteration; NSCLC, non-small cell lung cancer; RE, rearrangement; SV, short variant.

Supplementary figure 2: Type of GA in 10 DNA repair genes (FoundationOne®CDx assay only)

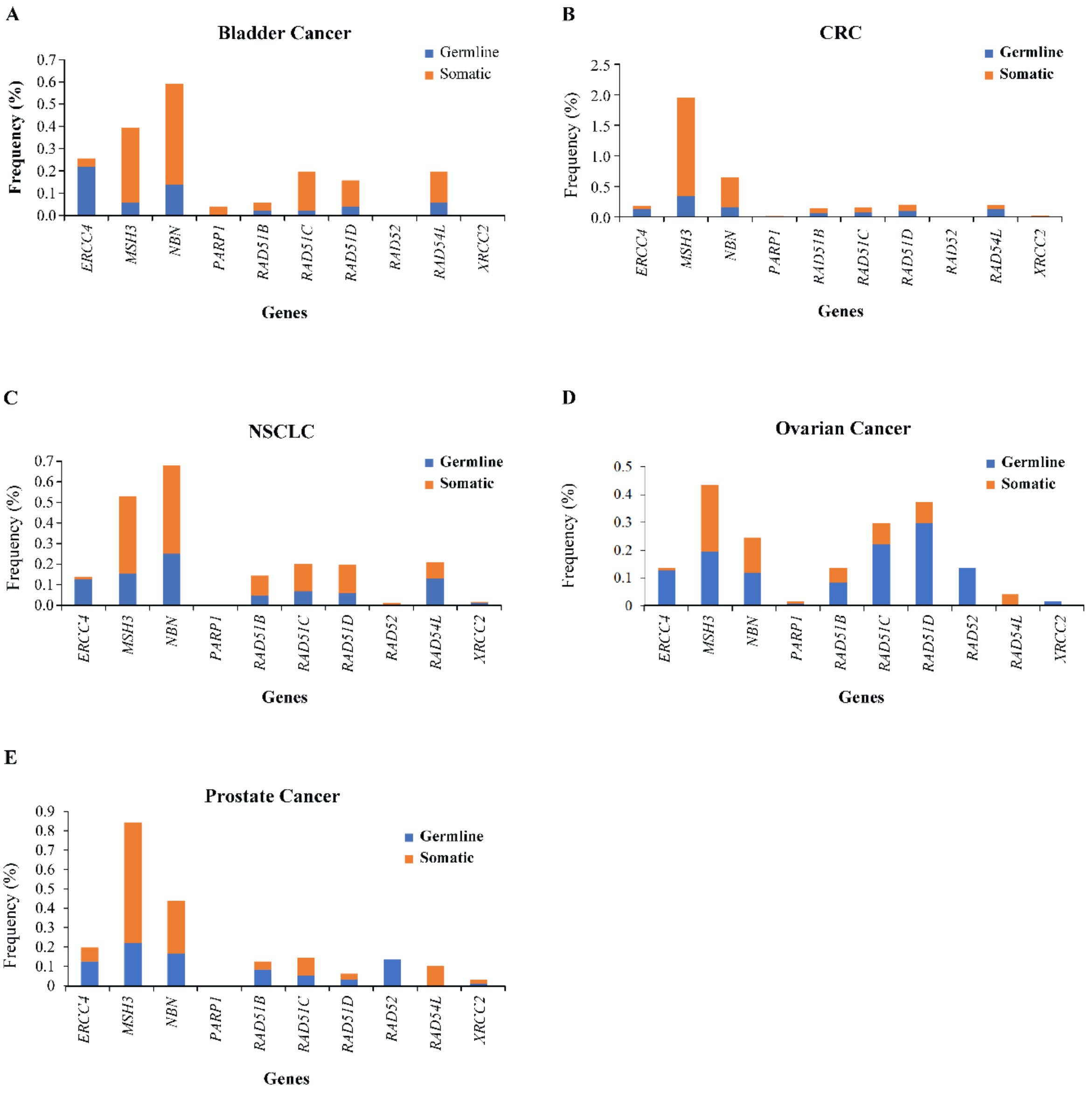

Mutations (SVs) in DNA repair genes were assessed for germline only or somatic only status (Panels A–E). CRC, colorectal cancer; GA, genomic alteration; NSCLC, non-small cell lung cancer.

Supplementary figure 3: Number of gene alterations in 10 DDR genes (FoundationOne®CDx assay only)

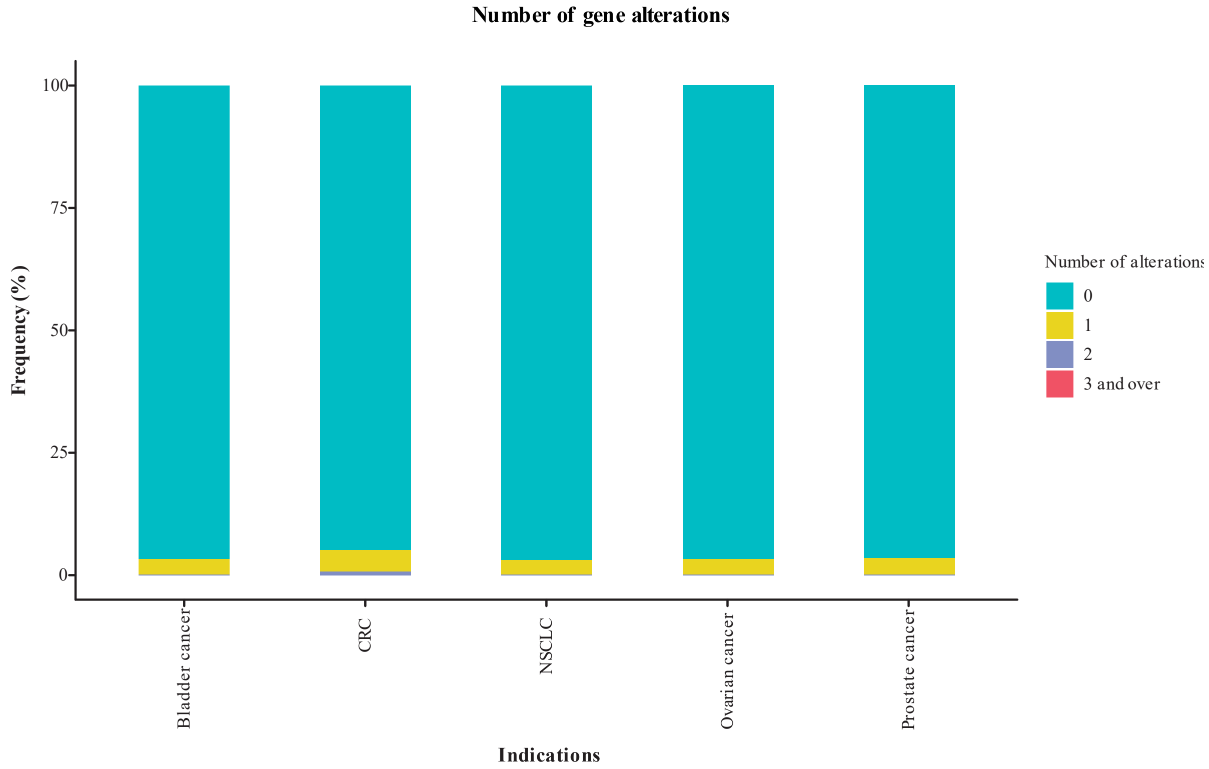

Number of DDR alteration	Bladder Cancer	CRC	NSCLC	Ovarian Cancer	Prostate Cancer
0	5444	26282	40243	12810	10808
1	181	1221	1238	420	384
2	6	207	49	18	18
>3	0	20	0	1	0

Number of GAs in DDR genes: 0: no alteration in DDR genes; 1: one alteration in DDR genes, 2: two alterations in DDR genes, >3: at least three alterations in DDR genes (could be same gene or multiple different genes). CRC, colorectal cancer; DDR, DNA damage response; GA, genomic alteration; NSCLC, non-small cell lung cancer.
