# Supplementary material for: Relationship among DDR gene mutations, TMB and PD-L1 in solid tumour genomes identified using clinically actionable biomarker assays
Source: NPJ Precis Oncol. 2023 Oct 11;7:103. doi: 10.1038/s41698-023-00442-4 (PMC10567713; doi:10.1038/s41698-023-00442-4)
Supplement: Supplementary file 2 — Reporting summary [file 41698_2023_442_MOESM2_ESM.pdf]

nature research

Corresponding author(s): Dr. Zheng Feng

Reporting Summary

Nature Research wishes to improve the reproducibility of the work that we publish. This form provides structure for consistency and transparency in reporting. For further information on Nature Research policies, see Authors & Referees and the Editorial Policy Checklist.

Statistical parameters

When statistical analyses are reported, confirm that the following items are present in the relevant location (e.g. figure legend, table legend, main text, or Methods section).

n/a	Confirmed	
☐	☒	The exact sample size (n) for each experimental group/condition, given as a discrete number and unit of measurement
☐	☒	An indication of whether measurements were taken from distinct samples or whether the same sample was measured repeatedly
☐	☒	The statistical test(s) used AND whether they are one- or two-sided<br>*Only common tests should be described solely by name; describe more complex techniques in the Methods section.*
☐	☒	A description of all covariates tested
☐	☒	A description of any assumptions or corrections, such as tests of normality and adjustment for multiple comparisons
☐	☒	A full description of the statistics including central tendency (e.g. means) or other basic estimates (e.g. regression coefficient) AND variation (e.g. standard deviation) or associated estimates of uncertainty (e.g. confidence intervals)
☐	☒	For null hypothesis testing, the test statistic (e.g. F, t, r) with confidence intervals, effect sizes, degrees of freedom and P value noted<br>*Give P values as exact values whenever suitable.*
☒	☐	For Bayesian analysis, information on the choice of priors and Markov chain Monte Carlo settings
☒	☐	For hierarchical and complex designs, identification of the appropriate level for tests and full reporting of outcomes
☐	☒	Estimates of effect sizes (e.g. Cohen's d, Pearson's r), indicating how they were calculated
☐	☒	Clearly defined error bars<br>*State explicitly what error bars represent (e.g. SD, SE, CI)*

Our web collection on statistics for biologists may be useful.

Software and code

Policy information about availability of computer code

Data collection	No software was used for data collection. We did not collect our own data for this study. This study utilized genomic results from solid tumor cases sequenced with the FoundationOne and FoundationOneCDx assays during routine clinical care
Data analysis	All statistical analyses were performed using the R (v4.0.3) and Python (v2.7.6) software packages

For manuscripts utilizing custom algorithms or software that are central to the research but not yet described in published literature, software must be made available to editors/reviewers upon request. We strongly encourage code deposition in a community repository (e.g. GitHub). See the Nature Research guidelines for submitting code & software for further information.

Data

Policy information about availability of data

All manuscripts must include a data availability statement. This statement should provide the following information, where applicable:

- Accession codes, unique identifiers, or web links for publicly available datasets
- A list of figures that have associated raw data
- A description of any restrictions on data availability

The authors declare that all relevant aggregate data supporting the findings of this study are available within the article and its supplementary information files. In accordance with the Health Insurance Portability and Accountability Act, we do not have IRB approval or patient consent to share individualized patient genomic

data, which contains potentially identifying or sensitive patient information and cannot be reported in a public data repository. Foundation Medicine is committed to collaborative data analysis and has well established and widely used mechanisms by which qualified researchers can query our core genomic database of >500,000 de-identified sequenced cancers. More information and mechanisms for data access can be obtained by contacting the corresponding author or the Foundation Medicine Data Governance Council at data.governance.council@foundationmedicine.com

Field-specific reporting

Please select the best fit for your research. If you are not sure, read the appropriate sections before making your selection.

☒ Life sciences ☐ Behavioural & social sciences

For a reference copy of the document with all sections, see nature.com/authors/policies/ReportingSummary-flat.pdf

Life sciences

Study design

All studies must disclose on these points even when the disclosure is negative.

Sample size	This was a retrospective study utilizing the FMI genomic database of cancer cases submitted for CGP during routine clinical care. As such, no methods were used to predetermine sample size (n).
Data exclusions	Duplicate patient samples were excluded from the cohort as to limit the analysis to a unique sample per patient.
Replication	Not applicable for this study
Randomization	Not applicable for this study
Blinding	Not applicable for this study

Materials & experimental systems

Policy information about availability of materials

n/a	Involved in the study	
☒	☐	Unique materials
☐	☒	Antibodies
☒	☐	Eukaryotic cell lines
☒	☐	Research animals
☐	☒	Human research participants

Antibodies

Antibodies used	DAKO PD-L1 IHC 22C3 pharmDx
Validation	The PD-L1 22C3 antibody is validated for use in NSCLC patients to determine eligibility for pembrolizumab

Human research participants

Policy information about studies involving human research participants

Population characteristics	For Foundation Medicine (FMI) genomic analysis, approval for this study, including a waiver of informed consent and a HIPAA waiver of authorization, was obtained from the WCG Institutional Review Board (IRB; Protocol No. 20152817). The IRB granted a waiver of informed consent under 45 CFR § 46.116 based on review and determination that this research meets the following requirements: (i) the research involves no more than minimal risk to the subjects; (ii) the research could not practicably be carried out without the requested waiver; (iii) the waiver will not adversely affect the rights and welfare of the subjects.

Method-specific reporting

n/a	Involved in the study	
☒	☐	ChIP-seq
☒	☐	Flow cytometry
☒	☐	Magnetic resonance imaging
